# Genetic Diversity of Mitochondrial DNA of Bemisia tabaci (Gennadius) (Hemiptera: Aleyrodidae) Associated with Cassava and the Occurrence of Cassava Mosaic Disease in Zambia

**DOI:** 10.3390/insects11110761

**Published:** 2020-11-05

**Authors:** Patrick Chiza Chikoti, Mathias Tembo, James Peter Legg, Rudolph Rufini Shirima, Habibu Mugerwa, Peter Sseruwagi

**Affiliations:** 1 Zambia Agriculture Research Institute, Mt. Makulu Research Station, Private Bag 7, Chilanga 10101, Zambia; mathiastembo2002@yahoo.com; 2 International Institute of Tropical Agriculture, P.O. Box 34441 Dar es Salaam, Tanzania; j.legg@cgiar.org (J.P.L.); r.shirima@cgiar.org (R.R.S.); 3 Department of Entomology, University of Georgia, 1109 Experiment Street, Griffin, GA 30223, USA; mugerwahabibu@yahoo.com; 4 Mikocheni Agricultural Research Institute, P.O. Box 6226 Dar es Salaam, Tanzania; psseruwagi@yahoo.co.uk

**Keywords:** whitefly, genetic diversity, *mtCOI*, *Manihot esculenta*, CMD, Southern Africa

## Abstract

**Simple Summary:**

*Bemisia tabaci* is an important vector that transmits cassava brown streak viruses and cassava mosaic begomoviruses that cause cassava brown streak and cassava mosaic diseases, respectively. In 2013 and 2015 we carried out a study to determine the genetic variability within the *Bemisia tabaci* complex associated with cassava in Zambia. This investigation made use of mitochondrial cytochrome oxidase I gene sequences of samples collected from selected provinces of Zambia. We found three population subgroups (SGs): SSA1-SG1, SSA1-SG2 and SSA1-SG3 within the sub-Saharan Africa 1 (SSA1) genetic group. Whitefly abundance and the incidence of cassava mosaic disease were both greatest in Western Province, in which the SSA1-SG1 subgroup predominated. Establishing which genetic groups and populations of the *B. tabaci* species complex are associated with cassava mosaic disease and their distribution in the country is key to guiding the strategic deployment of resources to monitor disease spread and ensure food security for millions of cassava-dependent households.

**Abstract:**

*Bemisia tabaci* is an important vector of cassava brown streak viruses and cassava mosaic begomoviruses, the causal agents of cassava brown streak disease and cassava mosaic disease (CMD), respectively. A study was carried out to determine the genetic variability of *B. tabaci* associated with cassava and the occurrence of CMD in Zambia in 2013 and 2015. Phylogenetic analysis showed the presence of only the sub-Saharan Africa 1 (SSA1) genetic group in Zambia. The SSA1 population had three population subgroups (SGs): SSA1-SG1, SSA1-SG2 and SSA1-SG3. All three SSA1 population subgroups occurred in Western Province. However, only SSA1-SG3 occurred in Eastern Province, while only SSA1-SG1 occurred in North Western and Luapula Provinces. Adult *B. tabaci* were most abundant in Western Province in 2013 (11.1/plant) and 2015 (10.8/plant), and least abundant (0.2/plant) in Northern Province in both 2013 and 2015. CMD was prevalent in all seven provinces surveyed, with the highest incidence recorded in Lusaka Province in both 2013 (78%) and 2015 (83.6%), and the lowest in Northern Province in both 2013 (26.6%) and 2015 (29.3%). Although SSA1-SG1 occurred at greater abundances than the other subgroups, there was no direct association demonstrated between whitefly subgroup and incidence of CMD. Establishing which *B. tabaci* genetic groups and populations are associated with CMD and their distribution in the country is a key factor in guiding the development of CMD control strategies for cassava-dependent households.

## 1. Introduction

*Bemisia tabaci* (Gennadius) (Hemiptera: Aleyrodidae) is one of the world’s most important agricultural insect pests, attacking a wide range of crop hosts and causing considerable yield loss [1]. It is also a vector of more than 200 plant viruses [2,3]. Most of these viruses belong to the genus *Begomovirus*, one of the major groups of plant viruses [4]. *Bemisia tabaci* has a high degree of intraspecific biological and genetic variability [5]. The intraspecific variation and wide host range pose the greatest challenge to the control of whiteflies [6]. Among both juvenile and adult whitefly, no morphological character can be used to distinguish different members of the *B. tabaci* species complex [7,8]. However, research has revealed the existence of morphologically indistinguishable populations exhibiting different biological traits within *B. tabaci* [9,10,11]. These traits include virus transmission efficiency [3,12], host range [13,14], and adaptability [15,16].

In recent years, more reproducible and informative methods have become available to determine the genetic affiliation of *B. tabaci* populations. The most widely used example is the sequencing of portions of the mitochondrial cytochrome oxidase I (*mtCOI*) gene [17,18] and the use of consensus sequences to assign group affiliations [19]. Accurate identification of insects that are pests and/or virus vectors is a prerequisite for their effective management in order to reduce crop damage [4]. Genome-wide studies using single nucleotide polymorphisms (SNPs) have also been used to characterize genetic diversity among cassava-colonizing *B. tabaci* in Africa [20,21].

Cassava brown streak viruses and cassava mosaic begomoviruses are the causal agents of cassava brown streak disease (CBSD) and cassava mosaic disease (CMD), respectively, and are transmitted by *B. tabaci* [22,23,24]. These two diseases have severely impacted cassava (*Manihot esculenta* Crantz) in sub-Saharan Africa (SSA) [25]. About USD 1.9–2.7 billion is lost annually to CMD alone in Africa [25,26,27]. In Zambia, yield losses attributed to CMD were estimated at USD 51.7 million [28]. In addition to transmitting viruses, *B. tabaci* also physically damages cassava plants through excessive feeding and impairment of photosynthesis by facilitating the growth of sooty mold on the deposits of honeydew left on the leaves, which is especially problematic when high infestations of whitefly occur. In East Africa, the pandemic of severe CMD that devastated cassava crops in the 1990s [29,30] was associated with high whitefly populations and two distinct *B. tabaci* genetic groups: sub-Saharan Africa 1 and 2 (SSA1 and SSA2) [11,31]. SSA2 was particularly prevalent in areas at and behind the epidemic “front” [31], while SSA1 primarily occurred ahead of the “front”. Subsequently, it was demonstrated that SSA1 was sub-divided into several phylogenetically distinct sub-groups (SSA1-sub-group 1 to SSA1 sub-group 5) [12,32] based on *mtCOI* sequences, and that SSA1-sub-group 1 (SSA1-SG1) appeared to be spatially and temporally associated with the severe CMD pandemic as it spread through East and Central Africa between the late 1990s and early 2000s. Limited testing of small numbers of samples from southern African countries, as part of a larger Africa-wide study, showed that SSA1-SG3 was present in Mozambique, Malawi, Madagascar and coastal East Africa, as well as Zambia [21].

Although known to occur on cassava for decades, there is inadequate information on the genetic variability and geographical distribution of *B. tabaci* genotypes in Zambia. This is information that is key to the sustainable management of cassava viral diseases (CMD and CBSD). Knowledge of the diversity and distribution of *B. tabaci* is a matter that has become urgent in view of the recent reported detection and presence of CBSD in the country [33]. Therefore, the aim of the study was to determine the genetic variability of cassava-associated *B. tabaci* and the associated CMD incidence in the key cassava-growing provinces in the country.

## 2. Materials and Methods

### 2.1. Study Area

Zambia experiences a tropical savannah climate with three seasons: a hot dry season (August–October); a hot wet season (November–April); and a cool dry season (May–July). Northern, North Western and Luapula Provinces are in agro-ecological region III and experience approximately 1000 mm and above of rainfall per annum. Western, Central, Lusaka and Eastern Provinces are in agroecological region II and receive 800–1000 mm annually. Whitefly abundance, CMD incidence and symptom severity were assessed in 245 and 200 cassava fields in 2013 and 2015, respectively. The field assessments were carried out in seven provinces, including: Central (36 & 29), Eastern (51 & 44), Luapula (47 & 28), Lusaka (24 & 14), North Western (16 & 17), Northern (40 & 43) and Western (31 & 25). Samples of *B. tabaci* were collected in Western, Eastern, North Western and Luapula Provinces for molecular characterization. CBSD was not assessed systematically in either 2013 or 2015, although survey teams were aware of CBSD symptoms and none were seen in any of the fields assessed.

### 2.2. Sampling Methodology

The protocol of Sseruwagi et al. [34] was followed to collect material for the investigation of CMD incidence and symptom severity, and whitefly abundance in January to March for both the 2013 and 2015 field surveys. Cassava fields were selected at regular intervals of 10–15 km, although this distance was greater in areas with few cassava fields. In each field, 30 plants of 3 to 6 months old were sampled along two diagonals running through the field along an ‘X’ shape [34]. CMD incidence was determined by counting the proportion of symptomatic plants out of the total (30) sampled per field [34]. CMD symptom severity was recorded for each plant using a 1–5 scale [35], in which 1 indicated the absence of disease symptoms; 2—mild chlorosis over the entire leaflet or mild distortion at the base of leaflets with only the remainder of the leaflets appearing green and healthy; 3—moderate chlorosis throughout the leaf, narrowing and distortion of the lower one-third of leaflets; 4—severe mosaic distortion of two-thirds of the leaflets and general reduction of leaf size; and 5—severe mosaic discoloration and/or distortion of the entire leaf and plant stunting.

*B. tabaci* adults were counted for each sampled plant on the five top-most leaves of the tallest shoot by gently turning the leaf to make the underside visible. Samples of *B. tabaci* adults were collected from cassava plants using an aspirator and placed in 2 mL plastic vials containing 70% ethanol for preservation. The geo-coordinates (latitude, longitude and altitude) were recorded for each location using a global positioning system (GPS) (Etrex, HC Sumit, Garmin International Inc., Olathe, KS, USA).

### 2.3. Extraction of Whitefly DNA, PCR and Sequencing

The DNA extraction protocol was followed for all whiteflies collected; the PCR treatment of samples from the 2013 and 2015 surveys differed in terms of the primer sets and protocol used. Therefore, we describe the two processes separately as follows.

#### 2.3.1. 2013 Samples

DNA was extracted from adult whiteflies and PCR was performed to amplify an 850 bp fragment of *mtCOI* according to Frohlich et al. [36]. The extraction was done in the molecular laboratory at the International Institute of Tropical Agriculture (IITA) in Dar es Salaam, Tanzania. One adult whitefly was drawn from each sample tube and placed on an inverted Petri dish covered with Parafilm. The insect was gently ground in 10 µL of extraction buffer to release total DNA. An additional 30 µL of extraction buffer was added and the contents mixed before transferring to a 1.5 mL tube incubated on ice. The extracted total DNA was then incubated in a water bath (Gesellschaft Für Labortechnik mbH, Burgwedel, Germany) at 65 °C for 15 min and at 95 °C for 10 min using a block heater (Grant QBD2, Grant Instruments Ltd., Shepreth, UK). The DNA was then centrifuged briefly for 5 s to pellet the debris. The contents collected in the supernatant were then stored at −20 °C until use.

PCR products of the *mtCOI* fragment (approximately 850 bp) were produced using the forward primer MT10/C1-J-2195 (5′-TTGATTTTTTGGTCATCCAGAAGT-3′) and the reverse primer MT12/TL2-N-3014 (5′-TCCAATGCACTAATCTGCCATATTA-3′) [37] using a thermocycler (Applied Biosystem, GeneAmp PCR System 9700, Foster City, CA, USA), under the following conditions: first cycle of denaturation at 94 °C for 2 min, followed by 35 cycles of denaturation at 94 °C for 30 s, annealing at 54 °C for 30 s, 72 °C for 1 min and final extension at 72 °C for 10 min. A total reaction mixture of 20 µL was made up of 12.54 µL of distilled water, 2 µL of PCR buffer, 2.5 µL of 25 mM MgCl_2_, 0.76 µL of 2 mM dNTPs, 1 µL of 10 mM primer mix, 0.2 µL of Taq DNA polymerase and 1.0 µL of DNA template. The PCR products were electrophoresed using a Midicell Primo electrophoretic gel system in 1% agarose gel stained in GelRed at 100 V for 30 min in gels buffered with 1× TAE buffer. The gel was visualized and photographed using the UVP imaging system (Biomapping Systems, Digidoc-IT, El Dorado Hills, CA, USA). The resulting 38 PCR products were sequenced in both the forward and reverse directions at Macrogen Inc., Rockville, MD, USA.

#### 2.3.2. 2015 Samples

DNA was extracted from individual adult whiteflies following the procedure of Walsh et al. [38]. One whitefly was drawn from a composite sample and placed into a 1.5 mL tube containing 25 µL of 10% Chelex solution. The whitefly was gently ground to release total DNA and an additional 25 µL of 10% Chelex solution was added to the tube. The 1.5 mL tube with the extract was incubated at 56 °C for 20 min in a water bath. The extracted DNA was further incubated at 100 °C for 5 min using a block heater. The contents were then transferred to new tubes and centrifuged at 12,000 rpm for 5 min to pellet the debris. The contents collected in the supernatant were then stored at −20 °C until use.

*MtCOI* sequences (approximately 850 bp) were amplified using the primers 2195Bt (5′-TGRTTTTTTGGTCATCCRGAAGT-3′) and C012/Bt-sh2 (5′-TTTACTGCACTTTCTGCC-3′) [14]. Primer sets of both Simon et al. [37] and Mugerwa et al. [14] amplified the same region, allowing easy comparison between the sequences produced. Although primer pair MT10/C1-J-2195 and MT12/TL2-N-3014 has been widely used in many parts of the world, problems arising from their use have been reported [39]. However, the primer set used in Mugerwa et al. [14] was more specific to *B. tabaci*.

The PCR was carried out with the same PCR conditions as in 2013 using a thermocycler (Applied Biosystem, GeneAmp PCR System 9700, CA, USA). PCR products were electrophoresed using a Midicell Primo electrophoretic gel system in 1% agarose gel stained in GelRed at 100 V for 30 min in gels buffered with 1× TAE buffer. The gel was visualized and photographed using the UVP, Biomapping Systems, Digidoc-IT, USA. The resulting 30 PCR samples were sequenced in both the forward and reverse direction at Source BioScience, Nottingham, UK.

### 2.4. Data Analysis

Following the method described in Sseruwagi et al. [34], disease incidence was calculated as the percentage of CMD-symptomatic plants per field. Disease symptom severity data were edited to remove the symptomless (healthy) counts (score 1) and analysis was conducted for the CMD-affected plants (score 2–5) per field. Adult whitefly population data were determined at plant level. Means were separated using a one-way analysis of variance using SPSS (version 20).

For all fields where whitefly sequences were obtained, the relationships between whitefly population subgroup, location (province) and whitefly abundance, as well as those between whitefly population subgroup and CMD incidence were examined with Kruskal–Wallis non-parametric ANOVA analyses using MedCalc 19.5.3 (MedCalc Software Ltd., Ostend, Belgium). The same software was used to investigate the association between whitefly abundance and CMD by calculating Pearson’s correlation coefficient. Analyses were conducted separately for 2013 and 2015 in addition to the data for the two years combined.

### 2.5. Phylogenetic and Sequence Analysis

Whitefly *mtCOI* sequences were edited manually to produce a consensus contig sequence of 705 bp for each individual whitefly using CLC Main Workbench version 7 (CLC Bio, Qiagen). This sequence portion was identical for both primer sets used, so the results of all sequences obtained were directly comparable. The edited sequences were aligned with reference whitefly sequences obtained from the National Center for Biotechnology Information (NCBI) (Table 1), using the Clustal W algorithm option available in the MEGA 7 program [40]. The edited contig sequences were aligned using MEGA 7 to infer a phylogenetic tree with the HKY+G+I model. To determine the confidence values for the grouping within a tree, a bootstrap analysis was performed using the maximum-likelihood procedure with a bootstrap setting of 1000 replicates. We also constructed a phylogenetic tree using the model based maximum likelihood (ML) analysis for the same dataset. We discovered that the Hasegawa-Kishino-Yano parameter with discrete Gamma distribution (HKY+G+I) was the best fit model for our dataset, having had the lowest Bayesian Information Criterion (BIC) value.

There were 68 final sequences obtained, which were cleaned manually, and the ends trimmed using Chromas Lite (version 2.1.1). The sequences were compared with those obtained from GenBank using the NCBI BLASTn program. After conducting pairwise analysis using Geneious R11 (https://www.geneious.com), a number of samples were found to be identical. From the identical samples, representative sequences were then selected, and used to construct a final phylogenetic tree and to generate evolutionary divergence using MEGA 7.

### 2.6. Population Genetic Analysis

To elucidate the genetic differences among the samples collected, the nucleotide sequences of all the isolates used in the present study were investigated. Population diversity indices based on the *mtCOI*, including number of haplotypes (h), polymorphic sites (S), average number of nucleotide differences (k), nucleotide diversity (Pi), and haplotype diversity (Hd) were estimated for each collection. The neutrality test for each subgroup population, including Tajima’s D [43] and Fu’s Fs [44] values, were also estimated. Sequence variation for each of the main haplotype groups detected was analyzed using DnaSP v6 [45].

## 3. Results

### 3.1. CMD Symptoms and Sooty Mold

**Figure 1:**
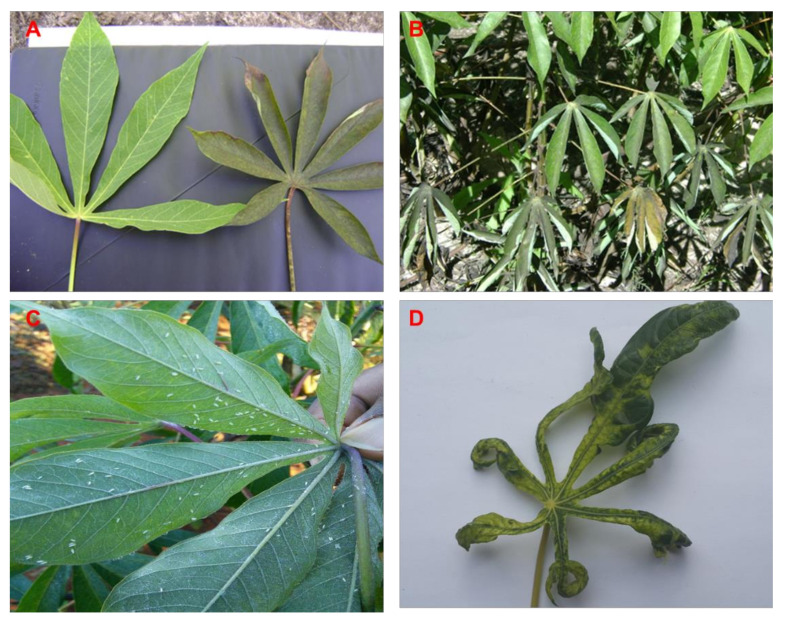
(**A**) Healthy leaf on the left and leaf with sooty mold on the right. (**B**) Cassava plant showing leaves with sooty mold. (**C**) Cassava leaves with *Bemisia tabaci*. (**D**) Cassava leaf showing severe symptoms of cassava mosaic disease.

CMD symptoms were observed in all provinces surveyed; however, the disease symptom expression depended on varieties cultivated (Supplementary Table S1). Generally, there were severe (leaf distortion, leaf narrowing and abscission) and mild mosaic (patchy green to yellow mosaic without leaf distortion) symptoms (Figure 1). In Western and North Western Provinces, sooty mold (unmeasured observation) occurred in both old (4–10 months after planting) and new fields (1–3 months after planting) (Figure 1).

### 3.2. Phylogenetic Analysis, Evolutionary Divergence and Geographical Distribution of B. tabaci mtCOI

**Figure 2:**
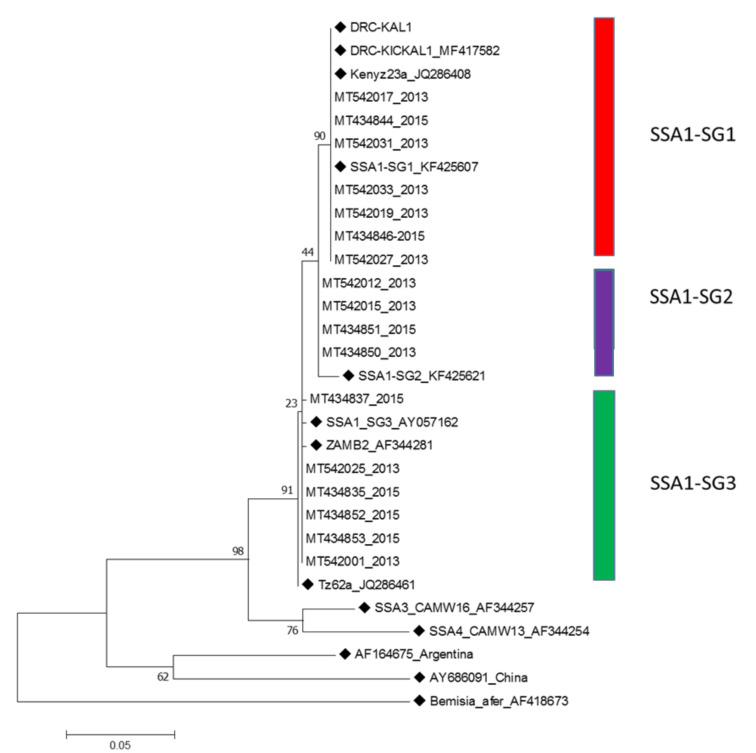
Maximum-likelihood phylogenetic tree constructed using mitochondrial cytochrome oxidase I sequences obtained from *Bemisia tabaci* collected on cassava in Zambia in 2013 and 2015. The tree is based on the Hasegawa-Kishino-Yano model with discrete gamma distribution (HKY + G + I) and 1000 bootstrap replications. Reference sequences from the National Center for Biotechnology Information are indicated with squares for comparison. *Bemisia afer* (AF418673) is included as an outgroup sequence. The numbers placed at each node indicate the bootstrap support for values >20.

**Figure 3:**
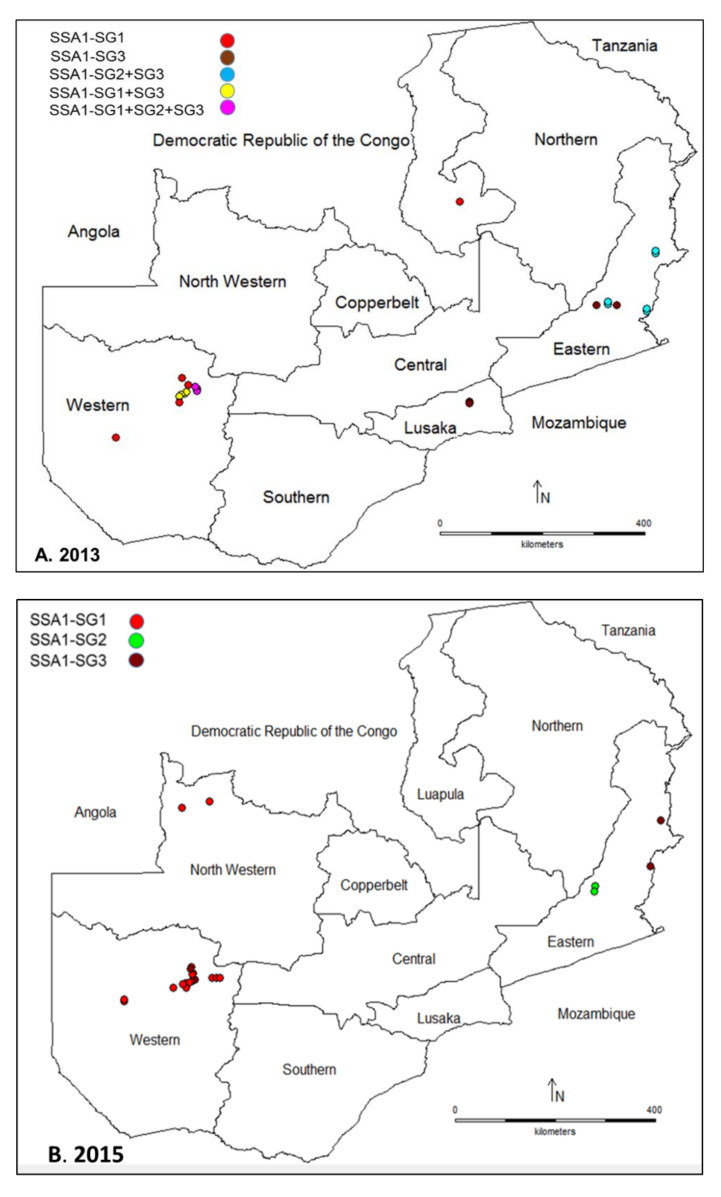
Distribution of *Bemisia tabaci* genetic groups sampled in Zambia. Each dot represents a site from which sampling was conducted and sequence obtained in (**A**) 2013 in Western, Luapula, Lusaka, and Eastern Provinces and (**B**) 2015 in North Western, Western and Eastern Provinces.

All the *B. tabaci* examined in this study clustered within the SSA1 genetic group. The sequence alignment followed by phylogenetic analysis grouped members of *B. tabaci* in three major clusters: SSA1-SG1, SSA1-SG2 and SSA1-SG3 (Figure 2). Out of the 38 *B. tabaci* sequences obtained in 2013, SSA1-SG1 accounted for 17 (44.7%), followed by SSA1-SG3 with 16 (42.1%) and SSA1-SG2 with five (13.2%) (Supplementary Table S2). In 2015, of the 30 sequences obtained, SSA1-SG1 accounted for 21 (70.0%), followed by SSA-SG3 with six (20.7%) and SSA1-SG2 with three (10.3%) (Supplementary Table S3). SSA1-SG1 was the most abundant subgroup in Western Province compared to SSA1-SG2 and SSA1-SG3, which mainly occurred in Eastern Province (Figure 3). The sequence data for 2013 (MT541997-MT542034) and 2015 (MT434827-MT434856) have been deposited in NCBI.

Among the 30 samples from the 2015 survey, SSA1-SG1 was the most abundant (n = 21, 70%). Most of the SSA1-SG1 clustered with DRC-KICKAL1 (MF417582) with nucleotide (nt) sequence homologies of 100% (Table 2). The rest had nt sequence similarity of 99.6% with SSA1-SG3 (AY057162) and 99.1% with SSA1-SG2 (KF425621) from Malawi and Burundi, respectively (Table 2). Evolutionary divergence among SSA1 subgroup sequences identified in this study ranged from 0.0–1.9% (Supplementary Table S4).

### 3.3. Diversity Indices

There was a very low level of polymorphism, estimated by haplotype diversity, amongst the three subgroups groups recorded in the study (Table 3). All eight sequences of SSA1-SG2 were identical, whilst SSA1-SG1 had four haplotypes and SSA1-SG3 had two. Haplotype diversity (Hd) for all the 68 sequences was found to be 0.039 SD. The average number of nucleotide differences (k) was calculated to be 5.53. For both SSA1-SG1 and SSA1-SG3, negative Tajima’s D values gave an indication of population expansion, although these values were not strong enough to be statistically significant in both cases.

### 3.4. CMD Incidence, Symptom Severity and Whitefly Abundance

There were notable significant differences between the provinces in 2013 with respect to CMD incidence (df = 6, F = 15.756, *p* < 0.0001), symptom severity (df = 6, F = 38.841, *p* < 0.0001) and whitefly abundance (df = 6, F = 268.908, *p* < 0.0001). The field situation was similar in 2015, with significant differences in CMD incidence (df = 6, F = 13.391, *p* < 0.0001), symptom severity (df = 6, F = 91.809, *p* < 0.000) and whitefly abundance (df = 6, F = 221.086, *p* < 0.0001). CMD was prevalent in all seven provinces surveyed (Table 4). Overall mean incidence for all the provinces was 50.5% in 2013 and 48.9% in 2015, with the highest incidences in Lusaka Province in 2013 (78.0%) and 2015 (83.6%), and lowest in Eastern Province with 26.6% in 2013 and 29.3% in 2015. CMD symptom severity was greatest in Lusaka Province (3.4 in 2013; 3.9 in 2015) and least in Eastern Province (2.8 in 2013; 3.1 in 2015). Adult whitefly numbers varied among the seven provinces. The populations were most abundant in Western Province in 2013 (11.1/plant) and 2015 (10.8/plant), and fewest in Northern Province with 0.2/plant in both years. In some fields in Western Province (Supplementary Tables S2 and S3) the number of whiteflies per plant exceeded 300, while in Northern and Eastern Provinces some fields had not a single whitefly. The abundance of whiteflies varied significantly between provinces in 2013 (H = 19.5; *p* = 0.00021), as well as for the combined data of 2013 and 2015 (H = 15.9; *p* = 0.0032), but differences were not statistically significant when considering 2015 alone (H = 0.7; *p* = 0.71).

There was a significant correlation between whitefly abundance and CMD incidence when considering data for both 2013 and 2015 (r = 0.28; *p* = 0.025), but no significant differences were apparent between SSA1 subgroups and CMD incidence, although in general it was apparent that CMD incidence was greater in Western Province where subgroup SSA1-SG1 predominated. Analysis of the relationship between subgroups and whitefly abundance showed that there were significant differences between abundances of subgroups when considering data for 2013 (H = 11.2; *p* = 0.0036) and both 2013 and 2015 (H = 6.8; *p* = 0.033), but differences were not significant when considering 2015 alone (H = 0.3; *p* = 0.86). In both cases where sub-groups had statistically different abundances, SSA1-SG1 was most abundant.

## 4. Discussion

We report the widespread occurrence of SSA1 as the main subgroup of *B. tabaci* occurring on cassava in Zambia. This study confirms previous reports by Berry et al. [10], although that study was based on limited sampling and there was no clear indication of the geographical location of the six samples studied. Three populations of SSA1 were identified on cassava: SSA1-SG1, SSA-SG2 and SSA1-SG3. In both 2013 and 2015, SSA1-SG1 was the dominant population compared to SSA1-SG2 and SSA1-SG3. The study by Berry et al. [10] identified only two populations of SSA1: SSA1-SG1 and SSA1-SG3. BLASTn comparisons of the six samples sequenced by Berry et al. [10] revealed that five of these were SSA1-SG1 and one was SSA1-SG3. The only other published *mtCOI* sequence of *B. tabaci* from Zambia was obtained from cassava in Chongwe, Lusaka Province, about 40 km east of Lusaka city [21]. This was also SSA1-SG3.

Populations of SSA1-SG1 have been reported most frequently from Tanzania, Uganda, Nigeria and Kenya [14,32,42,46,47,48] and were often associated with high whitefly abundance, particularly in East Africa. In our study, SSA1-SG1 occurred in areas with a relatively high whitefly abundance, such as Western and North Western Provinces, where populations were comparable to the high populations reported in East Africa [25,30]. Whiteflies on cassava have generally been observed to be more abundant in Western and North Western Provinces of Zambia [49,50,51], where SSA1-SG1 was also most prevalent. In this study, an average population exceeding 10 whiteflies per plant was recorded for both 2013 and 2015 in Western Province. To differentiate between low and high whitefly abundance, Legg [52] suggested a mean whitefly abundance threshold of ‘5/plant’. Using this definition, Western Province was categorized as having a ‘high abundance’ of whiteflies. Furthermore, the presence of sooty mold on cassava leaves in some fields is indicative of high whitefly abundance.

Whitefly abundance was also relatively high in both 2013 and 2015 in North Western Province compared to other provinces in this and past studies [50,51]. Whitefly abundance was low in Eastern, Central, Luapula and Northern Provinces, with averages in 2013 of only 1.0, 0.7, 0.5 and 0.2/plant, respectively. In 2015, there were few whiteflies in Eastern, Luapula and Northern Provinces (0.6, 0.3 and 0.2/plant, respectively). The generally low whitefly populations in the surveyed farmers’ fields could be attributed to several factors, including an unfavorable climate with a long cool dry season (May to August) which is followed by a hot dry season (September to November). Cassava mosaic disease was prevalent with moderate to high incidence in many surveyed fields. The high disease incidence was mainly attributed to the use of highly susceptible local cultivars and CMD-affected planting materials, although the significant correlation between whitefly abundance and CMD incidence demonstrated in our study confirms that whiteflies are a key factor in spreading the viruses causing CMD in Zambia. These findings corroborate earlier reports on CMD in the country [51,53], but suggest that the situation has changed from the 1990s when CMD incidence and severity were reported to be low in Western Province [49].

Subgroup SSA1-SG1 was prominent in Western Province, and has been reported to be dominant in parts of East and Central Africa affected by severe CMD and CBSD pandemics [32,42,48]. Although SSA1-SG1 has previously been associated with abundant populations of *B. tabaci* on cassava in East and Central Africa, it has also been shown to occur more widely, including in West Africa as far as Liberia [21], where no unusual levels of abundance have been reported. *B. tabaci* whiteflies from cassava in countries immediately neighboring Zambia have been reported in recent years, such as SSA1-SG1 from eastern and south-eastern Democratic Republic of Congo (DRC), SSA1-SG1, SSA1-SG2 and SSA1-SG3 from Malawi, and SSA1-SG3 from Mozambique [21,54]. In Tanzania, which borders Zambia to the north-east, SSA1-SG2 and SSA1-SG3 were found in areas unaffected by the severe CMD pandemic [55]. It can be speculated that the lack of SSA1-SG1 in the Eastern Province of Zambia could be contributing to the low CMD incidence recorded in this and other studies [50]. The reason why SSA1-SG2 and SSA1-SG3 occur in Eastern Province while SSA-SG1 is absent is not known, as no physical barriers exist that might limit the spread of SSA1-SG1. No statistically significant correlation was detected between the whitefly subgroups and CMD. However, the lack of a clear correlation between CMD incidence and *B. tabaci* subgroups could be attributed to a small sample size of sequenced whiteflies that was obtained for both 2013 and 2015 and it would certainly be important for future studies to assess larger sample sizes to ensure adequate representation of all subgroups occurring on cassava in the country. 

Genetic divergence analyses showed a low level of variation between SSA1-SG1, SSA1-SG2 and SSA1-SG3, which was comparable to other studies [42,55]. This small divergence suggests that they belong to the same genetic group, based on the widely applied 3.5% partial *mtCOI* gene sequence divergence threshold used for species-level designation within the *B. tabaci* species complex [19].

The predominant SSA1-SG1 haplotype in this study shared 100% sequence identity with an SSA1-SG1 haplotype from DRC [MF417582 (DRC-KICKAL1)] which was included here in the phylogenetic tree (Figure 2). Although SSA1-SG2 and SSA1-SG3 were not as frequent as SSA1-SG1 in both 2013 and 2015, they contributed to the *B. tabaci* diversity within SSA1. In addition, the negative and significant values obtained for Fu’s Fs statistic and Tajima’s D signified demographic expansionary changes for SSA1-SG1 and SSA1-SG3. SSA1-SG1 has been reported to be the prominent haplotype on cassava in north-western Tanzania, DRC, Uganda and Central African Republic, which has also been associated with spread of CMD [20,55,56]. With the high CMD incidence reported in this study in both 2013 and 2015 and in other previous studies [50], it is likely that SSA1-SG1 could be driving the high CMD incidence reported in Western Province.

Knowledge of the genetic diversity of *B. tabaci* in Zambia is of great practical importance in the light of the recent outbreak of CBSD in the country. Changes in whitefly population abundance were the key driver of the severe CMD and CBSD pandemics in East and Central Africa [27,32]. It is therefore anticipated that any changes in *B. tabaci* and abundance will impact cassava farmers through the spread of CMD and CBSD and cause physical damage through sap sucking and the growth of sooty mold on heavily infested plants. This highlights the importance of directing increased educational programs toward the effective and sustainable management of *B. tabaci* whiteflies on cassava and the viruses that they transmit.

## 5. Conclusions

The study provides important information on the genetic diversity of *B. tabaci* in Zambia. The findings will lead to better understanding of the *B. tabaci* genotypes colonizing cassava in Zambia. Although our results indicate no strong association between CMD and SSA1 subgroups, partly due to a small sample size, the relatively high *B. tabaci* abundance in Western Province appears to be an important factor in the high level of CMD incidence observed there in both 2013 and 2015.

## Figures and Tables

**Table 1:** Whitefly genotypes used in the phylogenetic analysis of mitochondrial cytochrome oxidase I sequences and their GenBank accession numbers.

Sequence Name	Country	GenBank Reference	Code (*mtCOI*)	Author
Texas B type	USA	AF164675	-	Unpublished
Bur2-2	Burundi	KF425621	SSA1-SG2	[32]
Tz10	Tanzania	KF425607	SSA1-SG1	[32]
21Malaw	Malawi	AY057162	SSA1-SG3	[31]
CAMW13	Cameroon	AF344254	SSA4	[10]
CAMW16	Cameroon	AF344257	SSA3	[10]
ChinaCQAY686091NB	China	AY686091	-	[41]
DRC-KICKAL1	DRC	MF417582	SSA1-SG1	[20]
Kenyz23a	Kenya	JQ286408	SSA1-SG1	[42]
Tz62a	Tanzania	JQ286461	SSA1-SG3	[42]
ZAMB1	Zambia	AF344280	SSA1-SG1	[10]
ZAMB2	Zambia	AF344281	SSA1-SG3	[10]
ZAMB6	Zambia	AF344284	SSA1-SG1	[10]
*Bemisia afer*	Uganda	AF418673	*Bemisia afer*	[24]

**Table 2:** Comparison of the selected genetic groups from Zambia to the sequences in GenBank.

Sequence Name	Province ^a^	Subgroup Name	Closest Relative	Sequence from GenBank	Country ^b^
			(Nt Identity%)		
MT542001 ^c^	Eastern	SSA1-SG3	99.6	AY057162	Malawi
MT542025 ^c^	Western	SSA1-SG3	99.6	AY057162	Malawi
MT434853 ^d^	Eastern	SSA1-SG3	99.6	AY057162	Malawi
MT434852 ^d^	Eastern	SSA1-SG3	99.6	AY057162	Malawi
MT434835 ^d^	Western	SSA1-SG3	99.6	AY057162	Malawi
MT542019 ^c^	Western	SSA1-SG1	100	KF425607	Tanzania
MT542031 ^c^	Western	SSA1-SG1	100	KF425607	Tanzania
MT542017 ^c^	Western	SSA1-SG1	100	KF425607	Tanzania
MT434844 ^d^	Western	SSA1-SG1	100	MF417582	DRC
MT434855 ^d^	North Western	SSA1-SG1	100	MF417582	DRC
MT434830 ^d^	Western	SSA1-SG1	100	MF417582	DRC
MT434846 ^d^	Western	SSA1-SG1	100	MF417582	DRC
MT434827 ^d^	Western	SSA1-SG1	100	MF417582	DRC
MT542012 ^c^	Eastern	SSA1-SG2	99.1	KF425621	Burundi
MT542015 ^c^	Western	SSA1-SG2	99.1	KF425621	Burundi
MT434851 ^d^	Eastern	SSA1-SG2	99.1	KF425621	Burundi
MT434850 ^d^	Eastern	SSA1-SG2	99.1	KF425621	Burundi

^a^ Province in Zambia from where the sequence was obtained. ^b^ Country origin of sequence deposited in GenBank. ^c^ Whitefly sequences obtained in 2013 in this study. ^d^ Whitefly sequences obtained in 2015 in this study.

**Table 3:** Population genetic analysis of *Bemisia tabaci* groups from Zambia: 2013 and 2015.

Parameter	All	SSA1-SG1	SSA1-SG2	SSA1-SG3
Sample size	68	38	8	22
Number of haplotypes	7	4	1	2
Polymorphic sites (S)	15	3	0	1
Average number of nucleotide differences (k)	5.52941	0.25462	0	0.09091
Nucleotide diversity (Pi)	0.00744	0.00034	0	0.00012
Haplotype diversity (Hd)	0.662	0.245	-	0.091
Variance of Hd	0.00154	0.00796	-	0.00655
Standard deviation of Hd	0.039	0.089	-	0.081
Theta per sequence	3.13195	0.71402	-	0.27432
Theta per site	0.00422	0.00096	-	0.00037
Fu’s Fs statistic	1.00136	−1.79006	-	−1.67803
Tajima’s D *p* for Tajima’s D	2.23673 *p* < 0.05	−1.42080 *p >* 0.10	*-*	−1.16240 *p >* 0.10

**Table 4:** Cassava mosaic disease (CMD) incidence, disease severity and whitefly population on cassava in seven provinces of Zambia in (a) 2013 and (b) 2015.

2013								2015						
Province	Number of Fields	CMD Incidence (%)	SE	Mean CMD Severity (1–5)	SE	Mean Whitefly Population	SE	Number of Fields	CMD Incidence (%)	SE	Mean CMD Severity (1–5)	SE	Mean Whitefly Population	SE
Central	36	57.9	4.027	3.3	0.25	0.7	0.035	29	70.2	3.211	3.8	0.026	1.3	0.084
Eastern	51	26.6	4.324	2.8	0.29	1.0	0.085	44	29.3	4.527	3.1	0.037	0.6	0.079
Luapula	47	49.9	4.102	3.2	0.19	0.5	0.034	28	46.2	4.915	3.2	0.037	0.3	0.035
Lusaka	24	78	4.914	3.4	0.42	6.6	0.628	14	83.6	7.759	3.9	0.038	3.7	0.463
North Western	16	55.8	8.271	3.3	0.33	2.2	0.311	17	63.6	7.765	3.4	0.04	2.2	0.408
Northern	40	39.1	4.571	3.1	0.25	0.2	0.045	43	33.2	4.698	3.4	0.027	0.2	0.038
Western	31	73.1	3.529	3.1		11.1	0.451	25	58.8	5.688	3.3	0.028	10.8	0.599
Mean		50.5		3.2		3.3			48.9		3.5		2.3	
SE		2.045		0.11		0.117			2.32		0.013		0.104	
*p*-value (5%)		*p* < 0.0001		*p* < 0.0001		*p* < 0.0001			*p* < 0.0001		*p* < 0.0001		*p* < 0.0001	

SE = standard error of the mean.

## Supplementary Materials

The following are available online at https://www.mdpi.com/2075-4450/11/11/761/s1: Table S1: Symptoms observed on cassava varieties in different selected locations in Zambia in 2013 and 2015; Table S2: Whitefly abundance, CMD incidence and severity and genetic subgroup determined in Zambia in 2013; Table S3: Whitefly abundance, CMD incidence and severity and genetic subgroup determined in Zambia in 2015; Table S4: Estimate of evolutionary divergence (expressed as percent nucleotide divergence) between partial mitochondrial cytochrome oxidase I (*mtCOI*) sequence representatives of *B. tabaci* identified on cassava in Zambia as conducted using Tajima-Nei model in MEGA 7.
