# Supplementary material for: Genetic Diversity of Mitochondrial DNA of Bemisia tabaci (Gennadius) (Hemiptera: Aleyrodidae) Associated with Cassava and the Occurrence of Cassava Mosaic Disease in Zambia
Source: Insects. 2020 Nov 5;11(11):761. doi: 10.3390/insects11110761 (PMC7694332; doi:10.3390/insects11110761)
Supplement: Supplementary file 1 [file insects-11-00761-s001.zip › insects-919413-insects-Supplementary Table/insects-911143 Supplementary Table 1.docx]

S1 Table: Symptoms observed on cassava varieties in different selected locations in Zambia in 2013 and 2015

| **Sn** | **Province** | **Latitude** | **longitude** | **Variety** | **Symptom*** |
| --- | --- | --- | --- | --- | --- |
| 1 | Western | -14.8244 | 24.6462 | Nalumino | M, LD, LN |
| 2 | Western | -14.7519 | 24.5761 | Nalumino | LD, LC, LN, M |
| 3 | Western | -14.6809 | 24.5319 | Mutembo | LN, LC, M |
| 4 | Western | -14.8699 | 24.6179 | Itezi-tezi | LC, LD, M |
| 5 | Western | -14.6422 | 24.5552 | Nalumino | LD, LN, LC, M |
| 6 | Western | -14.8349 | 24.9327 | Mweru | LN, LC, M |
| 7 | Western | -14.9313 | 24.4371 | Nalumino | LD, LN, M |
| 8 | Western | -14.9038 | 24.5481 | Itezi-tezi | LD, LN, M |
| 9 | Western | -14.9116 | 24.5195 | Nalumino | LN, LC, M |
| 10 | Western | -14.8297 | 25.0060 | Nalumino | LD, LC, LN |
| 11 | Western | -14.9434 | 24.3893 | Nalumino | LC, LN, M |
| 12 | Luapula | -10.8077 | 29.0508 | Beliate | LN, LC, M |
| 13 | Luapula | -9.47033 | 28.7358 | Musungu | LN, LC |
| 14 | Luapula | -9.70333 | 28.7700 | Mukulu | LC |
| 15 | Eastern | -13.2285 | 31.9378 | Manyopola | F, LD, M |
| 16 | Eastern | -12.8807 | 32.9516 | Manyopola | LD, F, M |
| 17 | Eastern | -12.0722 | 33.1468 | Manyopola | LD, M, P |
| 18 | North Western | -11.8458 | 24.3858 | Ndimbu | LN, LC, M |
| 19 | North Western | -11.7353 | 24.8853 | Nalumino | LC, M |
| 20 | North Western | -11.7353 | 24.8853 | Ndimbu | LC, M |
| 21 | Central | -13.5154 | 29.3572 | Mwakamoya | LN, LC |
| 22 | Central | -13.4934 | 29.3602 | Mwakamoya | LN, LC |
| 23 | Central | -13.5066 | 29.3657 | Bwelelako | LN, LC |
| 24 | Northern | -9.16434 | 32.2695 | Kasakata | LC |
| 25 | Northern | -9.61273 | 32.9719 | Lutundwe | M |
| 26 | Northern | -9.74166 | 31.1708 | Lyongwe | LD, M |

* M=mosaic, LD=leaf distortion, LN=leaf narrowing, LC: leaf chlorosis, F=filiform
