# Supplementary material for: Genetic Diversity of Mitochondrial DNA of Bemisia tabaci (Gennadius) (Hemiptera: Aleyrodidae) Associated with Cassava and the Occurrence of Cassava Mosaic Disease in Zambia
Source: Insects. 2020 Nov 5;11(11):761. doi: 10.3390/insects11110761 (PMC7694332; doi:10.3390/insects11110761)
Supplement: Supplementary file 1 [file insects-11-00761-s001.zip › insects-919413-insects-Supplementary Table/insects-911143Supplementary Table 2.docx]

Supplementary Table 2: Whitefly abundance, CMD incidence and severity and genetic subgroup determined in Zambia in 2013

| Way Point | Province | Latitude | Longitude | Total Whitefly number per field | Mean Whitefly population per field | CMD incidence | CMD severity | Subgroup |
| --- | --- | --- | --- | --- | --- | --- | --- | --- |
| 48 | Eastern | -13.33698 | 31.91925 | 114.00 | 3.80 | 80 | 1 | SG2 |
| 49 | Eastern | -13.33710 | 32.27424 | 491.00 | 16.37 | 96.7 | 1 | SG3 |
| 49 | Eastern | -13.33710 | 32.27424 | 491.00 | 16.37 | 96.7 | 1 | SG3 |
| 59 | Lusaka | -15.07132 | 29.63135 | 30.00 | 1.00 | 0 | 2.88 | SG1 |
| 59 | Lusaka | -15.07132 | 29.63135 | 30.00 | 1.00 | 0 | 2.88 | SG2 |
| 59 | Lusaka | -15.07132 | 29.63135 | 30.00 | 1.00 | 0 | 2.88 | SG3 |
| 71 | Lusaka | -15.10082 | 29.63507 | 32.00 | 1.07 | 43.3 | 3.1 | SG3 |
| 71 | Lusaka | -15.10082 | 29.63507 | 32.00 | 1.07 | 43.3 | 3.1 | SG3 |
| 71 | Lusaka | -15.10082 | 29.63507 | 32.00 | 1.07 | 43.3 | 3.1 | SG3 |
| 78 | Eastern | -13.32097 | 32.11703 | 21.00 | 0.70 | 0 | 2.73 | SG3 |
| 78 | Eastern | -13.27097 | 32.11703 | 21.00 | 0.70 | 0 | 2.73 | SG2 |
| 78 | Eastern | -13.27097 | 32.11703 | 21.00 | 0.70 | 0 | 2.73 | SG3 |
| 80 | Eastern | -12.40877 | 32.96915 | 35.00 | 1.17 | 50 | 2.73 | SG2 |
| 80 | Eastern | -12.35877 | 32.96915 | 35.00 | 1.17 | 50 | 2.73 | SG3 |
| 81 | Eastern | -13.39961 | 32.81103 | 96.00 | 3.20 | 90 | 2.56 | SG3 |
| 81 | Eastern | -13.44961 | 32.81103 | 96.00 | 3.20 | 90 | 2.56 | SG3 |
| 81 | Eastern | -13.39961 | 32.81103 | 96.00 | 3.20 | 90 | 2.56 | SG3 |
| 256 | Western | -14.83777 | 24.75911 | 61.00 | 2.03 | 43.3 | 2.46 | SG3 |
| 256 | Western | -14.88777 | 24.75911 | 61.00 | 2.03 | 43.3 | 2.46 | SG3 |
| 256 | Western | -14.80777 | 24.72911 | 61.00 | 2.03 | 43.3 | 2.46 | SG2 |
| 258 | Western | -14.77479 | 24.59477 | 268.00 | 8.93 | 70 | 2.90 | SG1 |
| 258 | Western | -14.77479 | 24.59477 | 268.00 | 8.93 | 70 | 2.90 | SG1 |
| 260 | Western | -14.65170 | 24.49429 | 1818.00 | 60.60 | 40 | 2.50 | SG1 |
| 260 | Western | -14.65170 | 24.49429 | 1818.00 | 60.60 | 40 | 2.50 | SG1 |
| 260 | Western | -14.65170 | 24.49429 | 1818.00 | 60.60 | 40 | 2.50 | SG1 |
| 263 | Western | -14.92243 | 24.54026 | 1593.00 | 53.10 | 93.3 | 3.28 | SG3 |
| 263 | Western | -14.89243 | 24.57026 | 1593.00 | 53.10 | 93.3 | 3.28 | SG1 |
| 263 | Western | -14.89243 | 24.57026 | 1593.00 | 53.10 | 93.3 | 3.28 | SG1 |
| 266 | Western | -14.94134 | 24.46936 | 562.00 | 18.73 | 53.3 | 2.88 | SG1 |
| 266 | Western | -14.97134 | 24.43936 | 562.00 | 18.73 | 53.3 | 2.88 | SG1 |
| 266 | Western | -14.97134 | 24.43936 | 562.00 | 18.73 | 53.3 | 2.88 | SG3 |
| 268 | Western | -15.08943 | 24.43623 | 175.00 | 5.83 | 56.7 | 2.94 | SG1 |
| 268 | Western | -15.08943 | 24.43623 | 175.00 | 5.83 | 56.7 | 2.94 | SG1 |
| 268 | Western | -15.08943 | 24.43623 | 175.00 | 5.83 | 56.7 | 2.94 | SG1 |
| 283 | Western | -15.72119 | 23.30663 | 287.00 | 9.57 | 73.3 | 3.00 | SG1 |
| 301 | Luapula | -11.47362 | 29.46008 | 73.00 | 2.43 | 93.3 | 2.89 | SG1 |
| 301 | Luapula | -11.47362 | 29.46008 | 73.00 | 2.43 | 93.3 | 2.89 | SG1 |
| 301 | Luapula | -11.47362 | 29.46008 | 73.00 | 2.43 | 93.3 | 2.89 | SG1 |
