# Supplementary material for: Genetic Diversity of Mitochondrial DNA of Bemisia tabaci (Gennadius) (Hemiptera: Aleyrodidae) Associated with Cassava and the Occurrence of Cassava Mosaic Disease in Zambia
Source: Insects. 2020 Nov 5;11(11):761. doi: 10.3390/insects11110761 (PMC7694332; doi:10.3390/insects11110761)
Supplement: Supplementary file 1 [file insects-11-00761-s001.zip › insects-919413-insects-Supplementary Table/insects-911143Supplementary Table 3.docx]

**Supplementary Table 3: Whitefly abundance, CMD incidence and severity and genetic subgroup determined in Zambia in 2015**

| Way Point | Province | Latitude | Longitude | Total Whitefly number per field | Mean Whitefly population per field | CMD incidence | CMD severity | Subgroup |
| --- | --- | --- | --- | --- | --- | --- | --- | --- |
| 100 | Western | -14.8244 | 24.6462 | 40 | 1.33 | 36.67 | 3.09 | SG1 |
| 102 | Western | -14.7519 | 24.5761 | 68 | 2.27 | 56.67 | 3.76 | SG1 |
| 105 | Western | -14.8699 | 24.6179 | 66 | 2.30 | 10.00 | 3.33 | SG3 |
| 104 | Western | -14.6422 | 24.5552 | 75 | 2.50 | 46.67 | 3.36 | SG1 |
| 117 | Western | -14.8349 | 24.9326 | 18 | 0.60 | 3.33 | 3.00 | SG1 |
| 97 | Western | -14.939 | 24.4197 | 211 | 7.03 | 66.67 | 3.25 | SG1 |
| 110 | Western | -14.9434 | 24.3893 | 787 | 26.20 | 70.00 | 3.33 | SG1 |
| 96 | Western | -14.9313 | 24.4371 | 599 | 19.97 | 80.00 | 3.13 | SG1 |
| 95 | Western | -14.9038 | 24.548 | 168 | 5.60 | 16.67 | 2.80 | SG1 |
| 118 | Western | -14.8297 | 25.006 | 4 | 0.13 | 0.00 | 1.00 | SG1 |
| 115 | Western | -15.2423 | 23.3131 | 132 | 4.40 | 66.67 | 3.10 | SG1 |
| 116 | Western | -15.2153 | 23.3194 | 356 | 11.87 | 93.33 | 3.18 | SG1 |
| 112 | Western | -15.0028 | 24.2268 | 1032 | 34.40 | 76.67 | 3.35 | SG1 |
| 98 | Western | -14.9712 | 24.4391 | 323 | 10.87 | 76.67 | 3.30 | SG1 |
| 99 | Western | -15.0155 | 24.4527 | 121 | 4.03 | 90.00 | 3.52 | SG1 |
| 109 | Western | -14.9116 | 24.5194 | 335 | 11.17 | 83.33 | 3.64 | SG1 |
| 118 | Western | -14.8297 | 25.006 | 4 | 0.13 | 0.00 | 1.00 | SG1 |
| 101 | Western | -14.766 | 24.5753 | 1335 | 44.50 | 76.67 | 3.52 | SG1 |
| 119 | Western | -14.8402 | 25.0687 | 6 | 0.20 | 0.00 | 1.00 | SG1 |
| 111 | Western | -14.963 | 24.3311 | 628 | 20.93 | 63.33 | 3.26 | SG3 |
| 103 | Western | -14.681 | 24.5319 | 1022 | 34.07 | 30.00 | 2.89 | SG3 |
| 110 | Western | -14.9434 | 24.3893 | 787 | 26.20 | 70.00 | 3.33 | SG1 |
| 226 | Eastern | -13.2285 | 31.9377 | 333 | 11.10 | 73.33 | 3.14 | SG2 |
| 225 | Eastern | -13.3119 | 31.9233 | 51 | 1.70 | 80.00 | 2.58 | SG2 |
| 227 | Eastern | -12.8807 | 32.9516 | 333 | 11.10 | 73.33 | 3.14 | SG3 |
| 243 | Eastern | -12.0722 | 33.1468 | 10 | 0.30 | 20.00 | 3.14 | SG3 |
| 270 | North Western | -11.8458 | 24.3858 | 62 | 2.07 | 40.00 | 3.33 | SG1 |
| 190 | North Western | -11.7353 | 24.8853 | 914 | 30.50 | 46.67 | 2.64 | SG1 |
