# Supplementary material for: Genetic Diversity of Mitochondrial DNA of Bemisia tabaci (Gennadius) (Hemiptera: Aleyrodidae) Associated with Cassava and the Occurrence of Cassava Mosaic Disease in Zambia
Source: Insects. 2020 Nov 5;11(11):761. doi: 10.3390/insects11110761 (PMC7694332; doi:10.3390/insects11110761)
Supplement: Supplementary file 1 [file insects-11-00761-s001.zip › insects-919413-insects-Supplementary Table/insects-911143Supplementary Table 4.docx]

Supplementary Table 4. Estimate of evolutionary divergence (expressed as percent nucleotide divergence) between partial mitochondrial cytochrome oxidase I (mtCOI) sequence representatives of *Bemisia tabaci* identified on cassava in Zambia as conducted using Tajima-Nei model^1^ in MEGA 7^2^.

|  |  | 1 | 2 | 3 | 4 | 5 | 6 | 7 | 8 | 9 | 10 | 11 | 12 | 13 | 14 | 15 | 16 | 17 | 18 | 19 | 20 | 21 | 22 | 23 |
| --- | --- | --- | --- | --- | --- | --- | --- | --- | --- | --- | --- | --- | --- | --- | --- | --- | --- | --- | --- | --- | --- | --- | --- | --- |
| 1 | MT542017_2013 | - | 0.0 | 0.3 | 0.3 | 0.1 | 0.0 | 0.1 | 0.0 | 0.9 | 0.9 | 0.9 | 0.9 | 1.7 | 1.4 | 1.4 | 1.6 | 1.4 | 1.4 | 1.4 | 1.6 | 7.3 | 8.1 | 29.3 |
| 2 | MT542031_2013 |  | - | 0.3 | 0.3 | 0.1 | 0.0 | 0.1 | 0.0 | 0.9 | 0.9 | 0.9 | 0.9 | 1.7 | 1.4 | 1.4 | 1.6 | 1.4 | 1.4 | 1.4 | 1.6 | 7.3 | 8.1 | 29.3 |
| 3 | MT542033_2013 |  |  | - | 0.3 | 0.1 | 0.3 | 0.1 | 0.3 | 1.1 | 1.1 | 1.1 | 1.1 | 2.0 | 1.7 | 1.7 | 1.9 | 1.7 | 1.7 | 1.7 | 1.9 | 7.2 | 8.1 | 29.3 |
| 4 | MT542019_2013 |  |  |  | - | 0.1 | 0.3 | 0.1 | 0.3 | 1.1 | 1.1 | 1.1 | 1.1 | 2.0 | 1.7 | 1.7 | 1.9 | 1.7 | 1.7 | 1.7 | 1.9 | 7.3 | 8.1 | 29.5 |
| 5 | MT542027_2013 |  |  |  |  | - | 0.1 | 0.0 | 0.1 | 1.0 | 1.0 | 1.0 | 1.0 | 1.9 | 1.6 | 1.6 | 1.7 | 1.6 | 1.6 | 1.6 | 1.7 | 7.2 | 8.0 | 29.3 |
| 6 | MT434844_2015 |  |  |  |  |  | - | 0.1 | 0.0 | 0.9 | 0.9 | 0.9 | 0.9 | 1.7 | 1.4 | 1.4 | 1.6 | 1.4 | 1.4 | 1.4 | 1.6 | 7.3 | 8.1 | 29.3 |
| 7 | MT434846_2015 |  |  |  |  |  |  | - | 0.1 | 1.0 | 1.0 | 1.0 | 1.0 | 1.9 | 1.6 | 1.6 | 1.7 | 1.6 | 1.6 | 1.6 | 1.7 | 7.2 | 8.0 | 29.3 |
| 8 | SSA1-SG1_Tz10_KF425607 |  |  |  |  |  |  |  | - | 0.9 | 0.9 | 0.9 | 0.9 | 1.7 | 1.4 | 1.4 | 1.6 | 1.4 | 1.4 | 1.4 | 1.6 | 7.3 | 8.1 | 29.3 |
| 9 | MT542012_2013 |  |  |  |  |  |  |  |  | - | 0.0 | 0.0 | 0.0 | 0.9 | 0.9 | 0.9 | 1.0 | 0.9 | 0.9 | 0.9 | 1.0 | 7.7 | 8.8 | 30.0 |
| 10 | MT542015_2013 |  |  |  |  |  |  |  |  |  | - | 0.0 | 0.0 | 0.9 | 0.9 | 0.9 | 1.0 | 0.9 | 0.9 | 0.9 | 1.0 | 7.7 | 8.8 | 30.0 |
| 11 | MT434851_2015 |  |  |  |  |  |  |  |  |  |  | - | 0.0 | 0.9 | 0.9 | 0.9 | 1.0 | 0.9 | 0.9 | 0.9 | 1.0 | 7.7 | 8.8 | 30.0 |
| 12 | MT434850_2015 |  |  |  |  |  |  |  |  |  |  |  | - | 0.9 | 0.9 | 0.9 | 1.0 | 0.9 | 0.9 | 0.9 | 1.0 | 7.7 | 8.8 | 30.0 |
| 13 | SSA1-SG2_Bu2-2_KF425621 |  |  |  |  |  |  |  |  |  |  |  |  | - | 1.7 | 1.7 | 1.9 | 1.7 | 1.7 | 1.7 | 1.9 | 8.3 | 8.8 | 30.0 |
| 14 | MT542025_2013 |  |  |  |  |  |  |  |  |  |  |  |  |  | - | 0.0 | 0.1 | 0.0 | 0.0 | 0.0 | 0.1 | 7.5 | 8.5 | 29.8 |
| 15 | MT542001_2013 |  |  |  |  |  |  |  |  |  |  |  |  |  |  | - | 0.1 | 0.0 | 0.0 | 0.0 | 0.1 | 7.5 | 8.5 | 29.8 |
| 16 | MT434837_2015 |  |  |  |  |  |  |  |  |  |  |  |  |  |  |  | - | 0.1 | 0.1 | 0.1 | 0.3 | 7.7 | 8.6 | 29.5 |
| 17 | MT434835_2015 |  |  |  |  |  |  |  |  |  |  |  |  |  |  |  |  | - | 0.0 | 0.0 | 0.1 | 7.5 | 8.5 | 29.8 |
| 18 | MT434852_2015 |  |  |  |  |  |  |  |  |  |  |  |  |  |  |  |  |  | - | 0.0 | 0.1 | 7.5 | 8.5 | 29.8 |
| 19 | MT434853_2015 |  |  |  |  |  |  |  |  |  |  |  |  |  |  |  |  |  |  | - | 0.1 | 7.5 | 8.5 | 29.8 |
| 20 | SSA1_SG3_21Malaw_AY057162 |  |  |  |  |  |  |  |  |  |  |  |  |  |  |  |  |  |  |  | - | 7.7 | 8.6 | 30.0 |
| 21 | SSA3_CAMW16_AF344257 |  |  |  |  |  |  |  |  |  |  |  |  |  |  |  |  |  |  |  |  | - | 7.0 | 30.5 |
| 22 | SSA4_CAMW13_AF344254 |  |  |  |  |  |  |  |  |  |  |  |  |  |  |  |  |  |  |  |  |  | - | 31.0 |
| 23 | *Bemisia afe*r_AF418673 |  |  |  |  |  |  |  |  |  |  |  |  |  |  |  |  |  |  |  |  |  |  | - |

1. Tajima F. and Nei M. (1984). Estimation of evolutionary distance between nucleotide sequences. Molecular Biology and Evolution 1:269-285.

2. Kumar S., Stecher G., Li M., Knyaz C., and Tamura K. (2018). MEGA 7: Molecular Evolutionary Genetics Analysis across computing platforms. Molecular Biology and Evolution 35:1547-1549.
